# Selective head cooling for the treatment of neurologic complications of acute liver failure in a newborn with disseminated herpes infection

**DOI:** 10.1186/2193-1801-2-572

**Published:** 2013-10-29

**Authors:** Gregory Hansen, Michele Grimason, James W Collins, Mark S Wainwright

**Affiliations:** Department of Pediatrics, Ruth D. & Ken M. Davee Pediatric Neurocritical Care Program, Ann & Robert H. Lurie Children’s Hospital of Chicago, Northwestern University Feinberg School of Medicine, Chicago, IL 60611 USA; Division of Neonatology, Ann & Robert H. Lurie Children’s Hospital of Chicago, Northwestern University Feinberg School of Medicine, Chicago, IL 60611 USA

**Keywords:** Acute liver failure, Transcranial doppler, EEG, Neonate, Hypothermia

## Abstract

**Background:**

Neurologic complications of pediatric acute liver failure (ALF) are a major determinant of outcome. Management of these complications, including increased intracranial pressure (ICP) is largely supportive. Although hypothermia is an effective treatment for perinatal asphyxia and is used to reduce ICP following traumatic brain injury, it has not been evaluated for neurologic complications of ALF in the newborn.

**Methods:**

Case report.

**Results:**

We present a case of neonatal herpes simplex virus (HSV)-associated ALF with profound neurologic impairment and increased ICP. The patient was treated with selective head cooling, and monitored with transcranial doppler (TCD) studies of cerebral blood flow velocity, and electroencephalograms (EEG). The duration of head cooling was influenced by absent diastolic flow on TCDs, which subsequently improved during hypothermia. Continuous EEGs captured subclinical seizures, which improved with antiepileptic medications. Her death was attributed to a massive pulmonary hemorrhage and a hypoxemic cardiac arrest secondary to significant coagulopathy.

**Conclusion:**

This case demonstrates that selective head cooling may attenuate increased ICP in neonatal encephalopathy, and that TCDs may guide management in the absence of invasive monitoring.

## Introduction

ALF in children is rare, and results in death or need for liver transplantation (LT) in nearly 50% of cases (Squires et al. 2006). Neurologic complications of pediatric ALF are a major determinant of outcome (Ciocca et al. 2008). Among children with ALF who develop hepatic encephalopathy (HE), fewer than 20% with HE survive without LT (Rivera-Penera et al. 1997). There are currently no specific therapies available to treat neurologic complications of ALF in children or adults.

Herpes simplex virus (HSV) infection is a common cause of ALF in newborns. In a recent international registry, infants ≤ 90 days accounted for nearly 18% of reported pediatric ALF cases (Sundaram et al. 2011). Mortality rates with viral etiologies were 64%, and HSV was the most common microbial identified (Sundaram et al. 2011). A small neonatal case series reported a mortality of 80% with ALF due to HSV infection, with most deaths occurring within five days of admission to a tertiary center (Verma et al. 2006).

Management of neurologic complications of ALF, including increased intracranial pressure (ICP) in children is largely supportive. In adults, hypothermia has been proposed as promising treatment modality for ALF with increased ICP (Stravitz & Larzen 2009). Hypothermia is a safe and effective treatment for perinatal asphyxia (Shankaran et al. 2005; Azzopardi et al. 2009; Gluckman et al. 2005; Lin et al. 2006), but has not been evaluated for the management neurologic complications of ALF in the newborn. We present a case of neonatal HSV-associated ALF with profound neurologic impairment and increased ICP, treated with selective head cooling (Gluckman et al. 2005) and monitored with transcranial doppler (TCD) studies of cerebral blood flow velocity (Aggarwal et al. 2008).

## Case report

A single term female weighing 2940 grams was born to a healthy gravida 3 mother via an uncomplicated spontaneous vaginal delivery. At day of life (DOL) 5, she was febrile to 38.6°C, jaundiced, and dehydrated, with marked hepatomegaly. Her initial lab abnormalities included: AST 9763 IU/L, ALT 2166 IU/L, ALP 211 IU/L, total bilirubin 5 mg/dL, direct bilirubin 1.8 mg/dL, PTT 96 seconds, platelets 89 thou/μL and ammonia of 151 μMol/L. Ampicillin, cefotaxime, acyclovir and vitamin K were administered.

**Figure 1:**
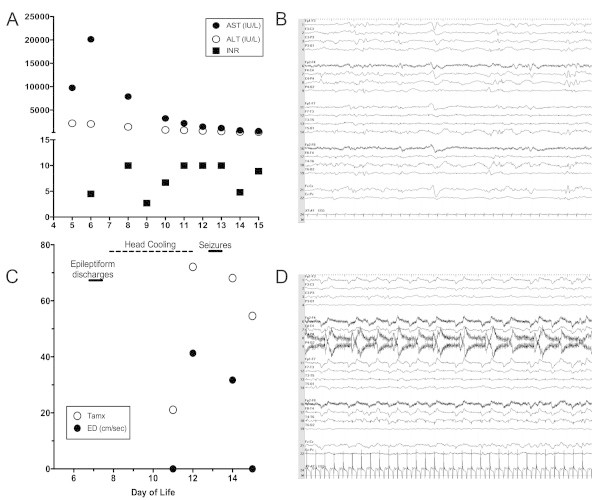
**Laboratory, TCD and EEG data. A**. Selected laboratory results showing changes in liver enzymes and synthetic function beginning with admission of day of life (DOL) 5. **B**, Continuous EEG on DOL7 showing focal bifrontal and bitemporal epileptiform discharges. **C**. Serial transcranial doppler results showing undetectable end-diastolic (ED) velocities in the middle cerebral artery on DOL 11 during hypothermia and DOL 14 after rewarming, with recovery of flow during head cooling on DOL 12 and early after re-warming on DOL 14. Time-averaged mean of the maximal velocities (Tamx) show initial reduction in velocity during cooling, followed by recovery. **D**, Continuous EEG on DOL13 showing right sided electrographic seizure. ALT, alanine aminotransferase; AST, aspartate aminotransferase; TCD, transcranial doppler.

Overnight, she became less responsive with several desaturations requiring transfer to our NICU and intubation. Admission exam showed a flat anterior fontanel and head circumference of 33.5 cm. Head ultrasound was unremarkable. Additional laboratory abnormalities included ferritin > 100 000 ng/L, INR 4.5, lactate 5 mEq/L, with a rising AST (Figure 1A). She was treated with IVIG (1 gm/kg) for suspected neonatal hemochromatosis. On DOL7, she became increasing oliguric and hypotensive, requiring treatment with dopamine. Rectal, perineal and mouth swabs became PCR positive for HSV. Routine EEG detected focal bifrontal and bitemporal epileptiform discharges, but no seizures [Figure 1B].

On DOL8 her exam was notable for widely spaced sutures, increased head circumference to 35 cm, decreased responsiveness and absent spontaneous movements. Her coagulopathy continued to worsen, with an INR > 10, and a PTT > 150 seconds. Although a head ultrasound was unremarkable, to reduce ICP she was treated with selective head cooling (goal systemic temperature 34.5-35.0°) for 92 hr using a modification of an established protocol for treatment of HIE (Gluckman et al. 2005). Following our institution guidelines for neurologic management of ALF, the head of bed was elevated to 30 degrees, L-carnitine (100 mg/kg/day IV) was given for 3 days, sodium maintained above 135 mEq/L, and mean arterial pressure above 50 mm Hg. Daily continuous (c)EEGs were performed for surveillance of non-convulsive seizures.

During the next 48 hours of cooling, she continued to require blood products with increased inotropic support for mean arterial pressures of 40 mm Hg and ongoing oliguria. Cooling did not induce bradycardia. Her neurologic exam was stable, and EEGs were attenuated and slow with infrequent bitemporal epileptiform discharges.

On DOL11 after 72 hours of cooling, she became diffusely hypotonic and areflexic with a stable head circumference of 35 cm. TCDs showed absent diastolic MCA flow (Figure 1C), suggesting increased ICP. The head of bed was increased to 40 degrees, and she remained cooled. The following morning, she moved extremities to stimulation, was coughing with suction and breathing above the ventilator, but continued to be hypotonic and areflexic. TCDs then showed diastolic flow suggesting a reduction in ICP. Both head ultrasound and EEG were unchanged. On DOL 12, following a total of 92 hours of cooling, she was rewarmed with a goal rate of of 0.2°C per hour.

The next morning, she had non-suppressible bilateral arm jerking, tachycardia and desaturations, which resolved with phenobarbital (Figure 1D). Her exam changed with arms flexed bilaterally, and an increased head circumference to 36.5 cm. EEG showed intermittent runs of vertex spikes, consistent with subclinical seizures. Maintenance phenobarbital was started, with daily levels. Shortly thereafter with an INR >10, she developed a pulmonary hemorrhage which ceased with high frequency oscillatory ventilation and FFP.

On DOL14, she was moving all extremities to stimuli, coughing, and breathing above the ventilator. TCD showed increased diastolic flow (Figure 1C). The following morning she had spontaneous movements with eye opening. The endotracheal tube became occluded from a clot, she desaturated to 30%, and MAPs dropped to the 20s. After 20 minutes of manual bagging, she returned to her baseline HFOV settings. Hours later, she had eye and head deviation to the left with tonic clonic movements which resolved with a Phenobarbital bolus. HC increased to 37.5 cm. Her EEG was markedly suppressed, and TCDs again showed no end diastolic flow. On DOL16, she had another pulmonary hemorrhage, became hypoxemic and bradycardic, and expired rapidly after life sustaining support was withheld at her mothers request.

## Discussion

To our knowledge, this is the first application of selective head cooling for management of HE in a newborn with ALF. The use of hypothermia is well established for the treatment of moderate or severe HIE (Shankaran et al. 2005; Azzopardi et al. 2009; Gluckman et al. 2005; Lin et al. 2006). While these protocols initiated therapy within 6 hours of delivery, we began treatment on DOL 8 based on the clinical evidence of increased ICP and the poor prognosis for survival in disseminated HSV infections in the newborn period.

Hypothermia has been proposed for treatment of HE in ALF in adults. Preliminary studies (Jalan et al. 2004; Jalan et al. 1999) suggested that hypothermia may be an effective treatment of uncontrolled ICP in adults with ALF. Subsequently, protocol driven management of increased ICP in adult ALF patients has included hypothermia as an optional treatment modality (Raschke et al. 2008; Tofteng et al. 2002).

There is extensive experience with therapeutic hypothermia in children with acute brain injuries. In traumatic brain injury, hypothermia does not appear to change functional outcome (Adelson et al. 2005), and may increase mortality (Hutchinson et al. 2008). Therapeutic hypothermia has been recommended following cardiac arrest in children (Nolan et al. 2008), but evidence from randomized controlled trials is lacking (Scholefield et al. 2013). Our patient was too unstable to undergo lumbar puncture to confirm the presence of encephalitis, and the risks and benefit of hypothermia to treat encephalitis without increased ICP are not known.

The biochemical and physiologic effects of therapeutic hypothermia are well characterized (Polderman 2009). In neonatal HIE, hypothermia is well tolerated, and mild bradycardia and raised liver enzyme concentrations have been reported (Gluckman et al. 2005). During cooling, our patient required increased inotropic support, ongoing blood products for coagulopathy and developed worsening lactic academia. She did not become bradycardic. The day following rewarming, she developed a significant pulmonary hemorrhage. The specific contribution of either ALF or hypothermia to each of these derangements is not clear, particularly in the context of a worsening coagulopathy and an INR > 10 prior to cooling.

Both whole-body and selective head cooling have been shown to be safe and effective in reducing neurologic morbidity after mild and moderate perinatal asphyxia (Shankaran et al. 2005; Azzopardi et al. 2009; Gluckman et al. 2005; Lin et al. 2006). We chose to use selective head cooling to avoid potential systemic complications of whole body cooling in a patient with severe coagulopathy and hypotension. There are insufficient data to speculate on specific benefits or risks of either approach for treating intracranial hypertension due to ALF in the neonate.

We used TCDs as a measure of increased ICP in this patient, as invasive monitoring of ICP is often impractical in coagulopathic patients with ALF, and the benefit of such monitoring is not proven. Recently, TCDs have been studied in pediatric traumatic brain injury (TBI) (Melo et al. 2011), neonatal sepsis (Basu et al. 2012), cardiopulmonary bypass (Wang et al. 2010) and extracorporeal membrane oxygenation (O’Brien & Hall 2013) as a surrogate for cerebral blood flow. Our patient initially exhibited absent diastolic MCA flow, which improved with another day of head cooling, and correlated with an improved clinical exam. The extent of compromise of CBF due to cerebral edema was not apparent from the clinical exam and the patient was too unstable to transport for imaging. TCDs therefore provided a non-invasive approach to identify increased ICP, and to assess response to therapies.

We interpreted the clinical exam with widely spaced sutures and bulging fontanelle as signs of increased ICP. The absence of flow during diastole on the initial TCD study supported this interpretation, based on generally accepted approaches to the interpretation of TCD results (Sloan et al. 2004). However, it should be noted that these data have not been validated in neonates. In children, studies validating TCD results based on invasive recording of ICP are derived from TBI (Melo et al. 2011) and there is no comparable precedent in neonatal ALF.

We used serial cEEGs to monitor for non-convulsive seizures which may cause secondary neurologic injury. The presence of bitemporal epileptiform discharges with a positive serum PCR for HSV is consistent with encephalitis. The combination of cEEG and serial TCDs allowed us guide brain-directed therapy, including the use of anti-convulsant using non-invasive monitoring.

The mortality rate of infants with ALF is up to 58% (Sundaram et al. 2011). The progression of HE in the setting of ALF due to HSV infection is erratic but may evolve to seizures and refractory ICP (Verma et al. 2006). While this patient expired from pulmonary complications of coagulopathy and disseminated herpes infection, this case illustrates the potential for the use of selective head cooling for the treatment of ALF. Combined with TCDs and cEEG for non-invasive measurement of cerebral blood flow and detection of non-convulsive seizures, this approach offers promise to reduce the neurologic morbidity of ALF in one of the largest pediatric age groups.

## References

[CR1] Adelson PD, Ragheg J, Kanev P, Brockmeyer D, Beers SR, Brown SD, Cassidy LD, Chang Y, Levin H (2005). Phase II clinical trial of moderate hypothermia after severe traumatic brain injury in children. Neurosurgery.

[CR2] Aggarwal S, Brooks DM, Kang Y, Linden PK, Patzer JF (2008). Noninvasive monitoring of cerebral perfusion pressure in patients with acute liver failure using transcranial Doppler ultrasonography. Liver Transpl.

[CR3] Azzopardi DV, Strohm B, Edwards AD, Dyet L, Halliday HL, Juszczak E, Kapellou O, Levene M, Marlow N, Porter E, Thoresen M, Whitelaw A, Brocklehurst (2009). Moderate hypothermia to treat perinatal asphyxial encephalopathy. N Engl J Med.

[CR4] Basu S, Dewangan S, Shukla RC, Anupurva S, Kumar A (2012). Cerebral blood flow velocity in early-onset neonatal sepsis and its clinical significance. Eur J Pediatr.

[CR5] Ciocca M, Ramonet M, Cuarterolo M (2008). Prognostic factors in paediatric acute liver failure. Arch Dis Child.

[CR6] Gluckman PD, Wyatt JS, Azzopardi D, Ballard R, Edwards AD, Ferrier DM, Polin RA, Robertston CM, Thoresen M, Whitelaw A, Gunn AJ (2005). Selective head cooling with mild systemic hypothermia after neonatal encephalopathy: multicentre randomised trial. Lancet.

[CR7] Hutchinson JS, Ward RE, Lacroix J, Hebert PC, Barnes MA, Bohn DJ, Dirks PB, Doucette S, Fergusson D, Gottesman R, Joffe AR, Kirpalani HM, Meyer PG, Morris KP, Moher D, Singh RN, Skippen PW (2008). Hypothermia therapy after traumatic brain injury in children. N Engl J Med.

[CR8] Jalan R, O Damink SW, Deutz NE, Lee A, Hayes PC (1999). Moderate hypothermia for uncontrolled intracranial hypertension in acute liver failure. Lancet.

[CR9] Jalan R, Olde Damink SW, Deutz NE, Hayes PC, Lee A (2004). Moderate hypothermia in patients with acute liver failure and uncontrolled intracranial hypertension. Gastroenterology.

[CR10] Lin ZL, Yu HM, Lin J, Chen SQ, Liang ZQ, Zhand ZY (2006). Mild hypothermia via selective head cooling as neuroprotective therapy in term neonates with perinatal asphyxia: an experience from a single neonatal intensive care unit. J Perinatol.

[CR11] Melo JR, Rocco F, Blanot S, Cuttaree H, Sainte-Rose C, Oliveira-Filho J, Zerah M, Meyer PG (2011). Transcranial Doppler can predict intracranial hypertension in children with severe traumatic brain injuries. Childs Nerv Syst.

[CR12] Nolan JP, Neumar RW, Adrie C, Aibiki M, Berg RA, Bottiger BW, Callaway C, Clark RSB, Geocadin RG, Jauch EC, Kern KB, Laurent I, Longstreth WT, Merchant RM, Morely P, Morrison LJ, Nadkarni V, Peberdby MA, Rivers EP, Rodriguez-Nunez A, Selike FW, Spaulding C, Sunde K, Hoek TV (2008). Post-cardiac arrest syndrome: epidemiology, pathophysiology, treatment, and prognostication. A scientific statement from the International Liaison Committee On Resuscitation: the American Heart Association Emergency Cardiovascular Care Committee: the Council on Cardiovascular Surgery and Anesthesia; the Council on Cardiopulmonary, Perioperative, and Critical Care; the Council on Clinical Cardiology; the Council on Stroke (Part II). Resuscitation.

[CR13] O’Brien NF, Hall MW (2013). Extracorporeal membrane oxygenation and cerebral blood flow velocity in children. Pediatr Crit Care Med.

[CR14] Polderman KH (2009). Mechanisms of action, physiologic effects, and complications of hypothermia. Crit Care Med.

[CR15] Raschke RA, Curry SC, Rempe S, Gerkin R, Little E, Manch R, Wong M, Ramos A, Leibowitz AL (2008). Results of a protocol for the management of patients with fulminant liver failure. Crit Care Med.

[CR16] Rivera-Penera T, Moreno J, Skaff C (1997). Delayed encephalopathy in fulminant hepatic failure in the pediatric population and the role of liver transplantation. J Pediatr Gastroenterol Nutr.

[CR17] Scholefield B, Duncan H, Davies P, Gao Smith F, Khan K, Perkins GD, Morris K (2013). Hypothermia for neuroprotection in children after cardiopulmonary arrest. Cochrane Database Syst Rev.

[CR18] Shankaran S, Laptook AR, Ehrenkranz RA, Tyson JE, McDonald SA, Donovan EF, Fanaroff AA, Poole WK, Wright LL, Higgins RD, Finer NN, Carlo WA, Duara S, Oh W, Cotton M, Stevenson DK, Stoll BJ, Lemons JA, Guillet R, Jobe AH (2005). Whole-body hypothermia for neonates with hypoxic-ischemic encephalopathy. N Engl J Med.

[CR19] Sloan MA, Alexandrov AV, Tegeler CH, Spencer MP, Caplan LR, Feldmann E, Wechsler LR, Newel DW, Gomez DR, Babikian VL, Lefkowitz D, Goldman RS, Armon C, Hsu CY, Goodin DS (2004). Assessment: transcranial Doppler ultrasonography. Report of the Therapeutics and Technology Assessment Subcommittee of the American Academy of Neurology. Neurology.

[CR20] Squires RH, Shneider BL, Bucuvalas J (2006). Acute liver failure in children: the first 348 patients in the pediatric acute liver failure study group. J Pediatr.

[CR21] Stravitz RT, Larzen FS (2009). Therapeutic hypothermia for acute liver failure. Crit Care Med.

[CR22] Sundaram SS, Alonso EM, Narkewicz MR, Zhang S, Squires RH & Pediatric Acute Liver Failure Study Group (2011). Characterization and outcomes of young infants with acute liver failure. J Pediatrics.

[CR23] Tofteng F, Jorgensen L, Hansen BA, Ott P, Kondrup J, Larsen FS (2002). Cerebral microdialysis in patients with fulminant hepatic failure. Hepatology.

[CR24] Verma A, Dhawan A, Zuckerman M, Hadzic N, Baker AJ, Mieli-Vergani G (2006). Neonatal herpes simplex virus infection presenting as acute liver failure: prevalent role of herpes simplex virus type I. J Pediatr Gastroenterol Nutr.

[CR25] Wang W, Bai SY, Zhang HB, Bai J, Zhang SJ, Zhu DM (2010). Pulsatile flow improves cerebral blood flow in pediatric cardiopulmonary bypass. Artif Organs.

